# AI and the Future of Medical Countermeasures to Protect Against Biological Threats

**DOI:** 10.1093/ofid/ofag108

**Published:** 2026-03-03

**Authors:** Amesh A Adalja, Jaspreet Pannu, Thomas V Inglesby

**Affiliations:** Johns Hopkins Center for Health Security, Bloomberg School of Public Health, Baltimore, Maryland, USA; Johns Hopkins Center for Health Security, Bloomberg School of Public Health, Baltimore, Maryland, USA; Johns Hopkins Center for Health Security, Bloomberg School of Public Health, Baltimore, Maryland, USA

The development of medical countermeasures (MCMs) for biological threats has long operated at a structural disadvantage. Emerging infectious diseases are inherently unpredictable—events whose magnitude, geographic footprint, and clinical characteristics cannot be forecast with precision. Accidentally caused and deliberate biological threats likewise depend on uncertain and often opaque human actions. By contrast, developing MCMs relies on predictability: researchers and companies plan around defined biological endpoints, known populations, regulatory clarity, and steady demand. Government MCM programs such as Project BioShield, the Biomedical Advanced Research and Development Authority (BARDA), and Operation Warp Speed have tried to counter these challenges by underwriting risk and stabilizing demand, but the underlying uncertainty has remained [1].

Artificial intelligence (AI) has now emerged as a powerful engine for accelerating and optimizing the MCM development process. In this field, AI is no longer aspirational; it is operational. Researchers already use AI to accelerate monoclonal antibody design, identify stable antigenic targets, optimize binding affinities, screen millions of small molecules, and uncover previously hidden pathways in host–pathogen interactions. Beyond the wet-lab, AI supports supply chains, predictive maintenance, manufacturing scheduling, and clinical-trial optimization. Former Sanofi CEO Paul Hudson captured the scale of this shift when he noted that, in an industry where 90% of early-stage projects fail, AI is reducing preclinical discovery times by 30%–50% and lowering costs by 25%–50% [2].

Yet the translation of these capabilities fully into the biodefense sphere has been slower—not because AI's usefulness is in doubt, but because the ecosystem required for high-performing AI does not yet exist. AI systems are only as powerful as the data they are trained on. The datasets needed to train models that can meaningfully accelerate MCM development—high-quality pathogen sequence and function data, structural biology datasets, immunological profiles, negative results from failed experiments, and real-time outbreak data—are scattered across a patchwork of institutions. Proprietary datasets sit inside companies; NIH maintains intramural files and funds thousands of extramural labs whose data remain siloed; the Department of Defense and national laboratories hold sensitive or classified data; and smaller biotech firms lack infrastructure or access. As a result, one of the greatest chokepoints in applying AI to biothreat MCMs is not model architecture—it is data architecture.

Constructing such an architecture raises key questions: Who should house the data? Do some datasets warrant access and security controls? Who validates data-quality? What standards should govern interoperability? And how should proprietary information be protected while still enabling progress? Efforts such as the National Science Foundation's National Artificial Intelligence Research Resource (NAIRR) offer a partial answer by integrating public and private AI resources. But biodefense requires more than general AI infrastructure—it needs a purpose-built system organized around biological data and governed in a way that catalyzes faster, safer medical countermeasure development.

The National Security Commission on Emerging Biotechnology has recommended several concrete steps. These include authorizing the Department of Energy to build a Web of Biological Data as a single point of entry for high-quality biological datasets; establishing tiered security controls for misuse-relevant datasets; empowering the National Institute of Standards and Technology (NIST) to set national data standards to ensure biological data are AI-ready; and creating a federally supported network of automated “cloud labs” capable of generating high-quality, standardized experimental data at scale. Taken together, these initiatives would lay the foundations for a secure, interoperable, and resilient national bio-AI ecosystem [3].

International partners are beginning to build analogous systems. The Coalition for Epidemic Preparedness Innovations (CEPI) Pandemic Preparedness Engine is one of the most ambitious global attempts to operationalize rapid response by compressing vaccine development timelines to 100 days. The Engine links high-throughput antigen discovery, AI-enabled immunogen design, curated prototype-pathogen datasets, standardized data pipelines, and geographically distributed manufacturing capacity. CEPI's prototype-pathogen strategy—building deep datasets across entire viral families—is particularly relevant for AI because it enables machine learning systems to generalize effectively, identify conserved antigens, predict immune-escape trajectories, and generate vaccine candidates early in an outbreak. While CEPI operates internationally, its architecture offers a blueprint for how U.S. agencies that are tasked with responding to biothreats—BARDA, the Defense Advanced Research Projects Agency (DARPA), the Defense Threat Reduction Agency (DTRA), and the national laboratories—could create a domestically anchored but globally interoperable biodefense ecosystem.

The U.S. national laboratories are uniquely positioned to anchor and integrate such a ecosystem. Institutions such as Lawrence Livermore and Los Alamos combine high-performance computing, secure data environments, statistical and mechanistic modeling expertise, and wet-lab experimentation within a single organizational structure. They can host AI training environments capable of handling classified or sensitive datasets; curate pathogen and host datasets at scale; and build biological models to complement data-driven machine learning. Datasets could be generated by academia, government, or industry. The recently announced Genesis Mission Executive Order empowers the Department of Energy and the national labs to build an integrated AI platform for federal scientific datasets; biotechnology is highlighted, though it remains to be seen whether biodefense and pandemic preparedness will be prioritized within the effort [4].

Federal biodefense programs already illustrate what AI-enabled systems could deliver. The Department of Defense's Joint Program Executive Office for Chemical, Biological, Radiological, and Nuclear Defense (JPEO-CBRND) is developing the GUIDE program—Generative Unconstrained Intelligent Drug Engineering—to build libraries of preemptive MCM candidates, optimize them across multiple product characteristics, and position the United States to respond rapidly to engineered or unforeseen threats [5]. Academia and industry are likewise demonstrating the feasibility of computational antibody design that can co-optimize candidates across dozens of circulating and hypothetical viral variants. These are early indicators of what a fully interoperable bio-AI architecture could achieve if built at national scale.

Several chokepoints persist beyond data fragmentation. A major one is the continued reliance on animal models, particularly under the Food and Drug Administration's (FDA) Animal Rule, where human efficacy cannot be measured directly. Most animal systems are slow, expensive, and often poor surrogates for human immune responses. AI-enabled in silico models—multi-scale immune simulations, agent-based systems, and organ-on-chip data interpreted through machine learning—offer an opportunity to reduce or eventually replace animal testing. But regulatory pathways for evaluating AI-derived evidence remain underdeveloped. Building standards, validation frameworks, and data-quality criteria will require sustained collaboration among FDA, BARDA, DARPA, DTRA, and the national labs.

Another critical gap is the lack of standardized clinical data from lethal outbreaks such as Nipah virus or highly pathogenic avian influenza. These outbreaks frequently occur in remote or resource-limited regions where data collection is delayed, inconsistent, or dangerous. AI-enabled sensors, portable diagnostics with embedded analytics, and autonomous monitoring platforms could capture and transmit high-fidelity clinical and epidemiological data without requiring large field teams. This would both protect responders and generate the kind of continuous datasets needed for AI models to understand disease trajectories, host responses, and therapeutic windows.

A narrow subset of biodefense-relevant data is inherently dual-use. Some AI developers have already excluded human-infecting viruses from training data due to security concerns, and leading researchers have called for tiered access controls for pathogen data that could enable misuse [6]. Rather than continuing ad hoc practices, the community should identify datasets that are particularly misuse-enabling and secure them while allowing legitimate research through trusted research environments—similar to the OpenSAFELY model for human genomics data [7–9].

Taken together, the central chokepoints in AI-enabled MCM development fall into eight broad categories: fragmented data; inaccessible proprietary datasets; barriers to sharing data across civilian, defense, and intelligence agencies; inadequate secure compute infrastructure; limited trust and validation frameworks; insufficient regulatory pathways for in silico evidence; persistent workforce gaps; and dual-use risks. Addressing these challenges will require coordinated roles and sustained investment across the federal landscape.

In addition to these eight, it is also crucial to recognize that the responsible deployment of AI depends not only on data availability but also on robust ethical, legal, and safety frameworks. Lastly, it will be essential for non-U.S. MCM enterprises, such as those governed by U.K.'s Biosecurity Leadership Council and the E.U.'s Health Preparedness and Response Agency to be integrated and aligned.

The promise of AI in biodefense is not simply technological; it has the potential to reinvigorate a crucial health and national-security task. The essential ingredients already exist across the U.S. government, academia, industry, and international partners such as CEPI. The challenge—and opportunity—lies in integrating them into a coherent, preemptive biodefense architecture before the next biological threat emerges.
